# Molecular and ecological signatures of an expanding hybrid zone

**DOI:** 10.1002/ece3.4024

**Published:** 2018-04-16

**Authors:** Maren Wellenreuther, Jesús Muñoz, Jesús R. Chávez‐Ríos, Bengt Hansson, Adolfo Cordero‐Rivera, Rosa A. Sánchez‐Guillén

**Affiliations:** ^1^ Department of Biology Lund University Lund Sweden; ^2^ The New Zealand Institute for Plant & Food Research Ltd Nelson New Zealand; ^3^ Real Jardín Botánico (RJB‐CSIC) Madrid Spain; ^4^ Departamento de Biología Celular y Fisiología Unidad Periférica Tlaxcala Instituto de Investigaciones Biomédicas Universidad Nacional Autónoma de México Tlaxcala México; ^5^ Department of Ecology and Animal Biology E.E. Forestal University of Vigo Vigo Spain; ^6^ Instituto de Ecología A. C. Veracruz México

**Keywords:** hybrid zones, introgression, *Ischnura elegans*, niche shift, range expansion

## Abstract

Many species are currently changing their distributions and subsequently form sympatric zones with hybridization between formerly allopatric species as one possible consequence. The damselfly *Ischnura elegans* has recently expanded south into the range of its ecologically and morphologically similar sister species *Ischnura graellsii*. Molecular work shows ongoing introgression between these species, but the extent to which this species mixing is modulated by ecological niche use is not known. Here, we (1) conduct a detailed population genetic analysis based on molecular markers and (2) model the ecological niche use of both species in allopatric and sympatric regions. Population genetic analyses showed chronic introgression between *I. elegans* and *I. graellsii* across a wide part of Spain, and admixture analysis corroborated this, showing that the majority of *I. elegans* from the sympatric zone could not be assigned to either the *I. elegans* or *I. graellsii* species cluster. Niche modeling demonstrated that *I. elegans* has modified its environmental niche following hybridization and genetic introgression with *I. graellsii*, making niche space of introgressed *I. elegans* populations more similar to *I. graellsii*. Taken together, this corroborates the view that adaptive introgression has moved genes from *I. graellsii* into *I. elegans* and that this process is enabling Spanish *I. elegans* to occupy a novel niche, further facilitating its expansion. Our results add to the growing evidence that hybridization can play an important and creative role in the adaptive evolution of animals.

## 1 INTRODUCTION

Numerous species have recently undergone range expansions in response to anthropogenic changes and created de novo sympatric zones of overlap between formerly allopatric species (Canestrelli et al., 2016). The consequences of these newly created sympatric zones for species diversity are just beginning to be explored, and preliminary evidence is suggesting that they may trigger increased rates of introgressive hybridization. Such increased levels of species mixing are, in most cases, caused by a lack of reproductive barriers between the formerly allopatric species (Sánchez‐Guillén, Wellenreuther, & Cordero‐Rivera, 2012), which may have either been lost due to niche modification or secondarily through drift (Wellenreuther, Tynkkynen, & Svensson, 2010). While studies indicate that such de novo sympatric zones are commonly being created across a wide range of species, including invertebrates (Mallet, Wynne, & Thomas, 2011), vertebrates (Kelly, Whiteley, & Tallmon, 2010), and plants (Gómez, González‐Megías, Lorite, Abdelaziz, & Perfectti, 2015), efforts to explore the long‐term consequences of secondary sympatry on the genetic diversity and realized niche use are largely lacking. Indeed, recent research suggests that the known examples merely represent the tip of the iceberg and that future work will show that introgressive hybridization following range shifts is much more widespread than has been anticipated.

The outcome of species mixing via introgressive hybridization directly affects the genetic diversity of populations and is hence a critical determinant of the long‐term evolutionary population processes and patterns (Baack & Rieseberg, 2007; Ryan, Johnson, & Fitzpatrick, 2009). While such species mixing can be an important source of new genetic variation (Kays, Curtis, & Kirchman, 2009; Seehausen, 2004), it can also lead to genetic swamping and thus the genetic extinction of a population or even a species (Rhymer & Simberloff, 1996). Hybridization can also result in geographically limited hybrid zones with low levels of gene flow across otherwise stable population boundaries or may lead to reinforcement of reproductive barriers due to low‐hybrid fitness (Arnold, 1997). The majority of work thus far on these processes has focussed on plants (Rieseberg & Brunsfeld, 2012) and fishes (Keller et al., 2013; Seehausen, 2004), because hybridization in these groups is particularly common, but to a much lesser extent on invertebrates, with the exception of a few well‐documented cases of hybridizing non‐native species (Sánchez‐Guillén, Cordoba‐Aguilar, Hansson, Ott, & Wellenreuther, 2016).

In addition to effects on the genetic diversity, introgressive hybridization can also affect the ecological population dynamics of species via adaptive trait introgression, that is, the introduction of new alleles that increase the competitiveness of individuals (Arnold, 2004). Lewontin and Birch (1966) proposed that such adaptive trait introgression can facilitate the rate of adaptive evolution and gathered empirical evidence for this by studying the fruit fly *Drosophila tryoni* in Australia. This fly species has greatly extended its geographic range into warmer areas over the last 100 years, and Lewontin and Birch (1966) were able to demonstrate that the increase in upper temperature tolerance was caused by introgressive hybridization with *Drosophila neohumeralis*. Since then, it has become increasingly established that even though the initial hybridization is often deleterious, subsequent introgression can provide a unique opportunity for species to tap into genetic variation present in a closely related species and potentially take advantage of beneficial alleles (Baack & Rieseberg, 2007; Huerta‐Sanchez et al., 2014; Mallet, 2005; Rieseberg, 2009; Seehausen, 2004). It is noteworthy that alleles with widespread effects can spread particularly easy across species boundaries when species isolating barriers are incomplete (Coyne & Orr, 2004) to produce the signature of a trans‐species selective sweep (Brand, Kingan, Wu, & Garrigan, 2013).

Here, we study the hybrid zone of the blue‐tailed damselfly *Ischnura elegans* (Odonata: Coenagrionidae) in southern Europe, where it is overlapping with its sister species, the Iberian bluetail *Ischnura graellsii*. These species show only incomplete reproductive barriers, enabling chronic introgression (Sánchez‐Guillén et al., 2012). Specifically, we investigate the molecular and ecological signatures of this hybrid zone to (1) quantify the pattern and extent of molecular population divergence and introgression across populations inside and outside the hybrid zone, and 2) test if trait introgression has affected the ecological niche use of both species and its hybrids.

## 2 MATERIALS AND METHODS

### 2.1 Species natural history and ecology

*Ischnura elegans* has become an eco‐evolutionary model species because of its enigmatic female‐limited genetic color polymorphism and has been studied extensively for its population morph dynamics (Cordero & Andrés, 1996; Sánchez‐Guillén, Hansson, Wellenreuther, Svensson, & Cordero‐Rivera, 2011), the genetics of color (Chauhan, Wellenreuther, & Hansson, 2016; Chauhan et al., 2014; Sánchez‐Guillén, Van Gossum, & Cordero‐Rivera, 2005), and the processes maintaining the color polymorphism in nature (Sánchez‐Guillén et al., 2017; Svensson, Abbott, & Hardling, 2005; Takahashi, Yoshimura, Morita, & Watanabe, 2010). *Ischnura elegans* and *I. graellsii* are closely related (Sánchez‐Guillén et al., under revision) and share many phenotypic traits, including many preference traits for habitats. *Ischnura elegans* extends from Ireland to the Mediterranean and Japan, while *I. graellsii* extends across the western Mediterranean area including Iberia and Maghreb. Both species are common along running and standing water bodies, and both are tolerant to brackish waters (Sánchez‐Guillén, Hansson, et al., 2011). Whenever the habitat is suitable, both species form highly abundant populations and are commonly the dominant species in ponds and lakes. Previous studies have demonstrated a lack of isolation by distance in *I. elegans* and *I. graellsii* populations (Wellenreuther, Sánchez‐Guillén, Cordero‐Rivera, Svensson, & Hansson, 2011).

In the last years, *I. elegans* has also received attention because it is undergoing a range expansion in northern Europe and in Spain (Lancaster et al., 2016; Sánchez‐Guillén, Wellenreuther, Cordero‐Rivera, & Hansson, 2011). Despite its good dispersal ability, the distribution of *I. elegans* in Spain is discontinuous: It is common in north‐central Spain and along the Mediterranean coast, from Pyrenees to Murcia, where it is the dominant species. However, several populations of *I. elegans* were found in the early 1990s in north‐western Spain, more than 400 km away of the central Spanish populations (Ocharan Larrondo, 1987). Central Spain Earliest evidence for this hybridization came from work by Monetti, Sánchez‐Guillén, and Cordero‐Rivera (2002) who showed that allopatric *I. graellsii* are smaller in body size and have narrower and shorter wings and shorter tibiae, whereas *I*. *elegans* have a narrower space between the branches of each cercus and a greater distance between the branches of each paraproct. In sympatry, however, these morphological traits were intermediate, strongly suggesting that introgressive hybridization affects the overall phenotype (2002). Our preliminary work on the molecular population signatures in this hybrid zone showed evidence for extensive and asymmetric introgression (because of strong asymmetry in reproductive barriers, see Sánchez‐Guillén et al., 2012), with *I. elegans* replacing *I. graellsii* from many areas in Spain (Sánchez‐Guillén, Wellenreuther, et al., 2011).

### 2.2 Population sampling

**Figure 1:**
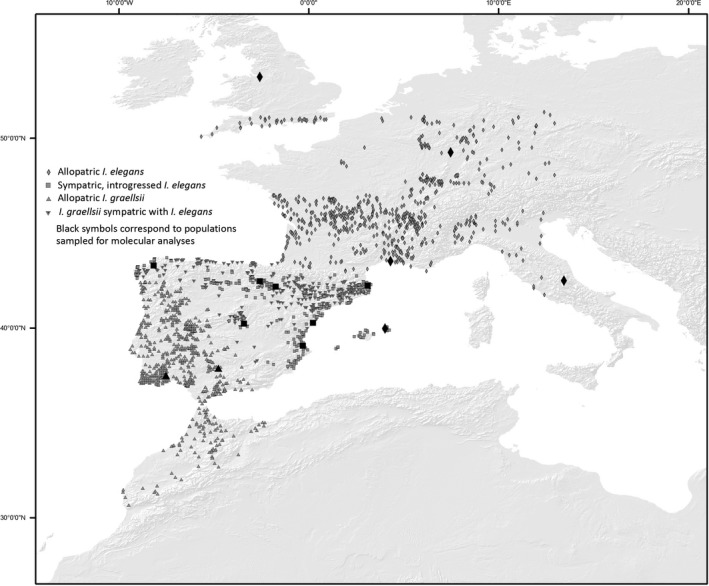
Map of sampled populations. Diamonds denote allopatric *Ischnura elegans*; squares sympatric, introgressed *I. elegans*; upside triangles allopatric *Ischnura graellsii*; and downside triangles: *I. graellsii* sympatric with *I. elegans*. Solid symbols correspond to populations sampled for molecular analyses

Molecular data were collected from 12 populations (Figure 1) of *I. elegans* and two populations of *I. graellsii*: seven *I. elegans* populations from the introgressed distribution (Doniños, Arreo, Baldajo, Alfaro, Estanyo de Europa, Amposta, and Marjal del Moro), five *I. elegans* populations from the allopatric distribution (Menorca, Vigueirat, Gran Sasso, Liverpool, and Kaiserslautern), and two *I. graellsii* populations from the allopatric distribution (Córdoba and Ribeira de Cobres). Population assignments were based on the individual morphology of damselflies caught at each location. Pure *I. elegans* are easily distinguished from *I. graellsii* based on the “promontory” trait, which is a structure located in the prothorax. Hybrids or introgressed individuals have intermediate structures between *I. elegans* and *I. graellsii*. Other characteristics such body size, color, and structure of the anal appendages are used in addition to this trait for visual identification for highly introgressed individuals. Previous molecular work using microsatellites has confirmed that morphological identification based on these traits is reliable (Sánchez‐Guillén, Hansson, et al., 2011).

### 2.3 DNA extraction and microsatellite genotyping

Genomic DNA for microsatellite typing was extracted from 14 populations (208 *I. elegans* individuals and 29 *I. graellsii*, Table 1) using a standard phenol/chloroform‐isoamyl alcohol protocol (Sambrook, Fritsch, & Maniatis, 1989). A total of 237 individuals were genotyped at 12 microsatellite markers (I‐026, I‐077, I‐058, I‐090, I‐129, I‐216, I‐002, I‐015, I‐041, I‐053, I‐095, and I‐134, Wellenreuther, Sánchez‐Guillén, Cordero‐Rivera, & Hansson, 2010). Polymerase chain reactions (PCRs) were carried out following the protocol in Wellenreuther, Sánchez‐Guillén, et al. (2010) and Wellenreuther, Tynkkynen, et al. (2010). Genotyping was conducted on an ABI PRISM 3730 Genetic Analyzer (Valencia University), and allele sizes were determined with GeneMapper 3.0 (both from Applied Biosystems).

**Table 1:** Population and country names that were included in the microsatellite work. Species are denoted as Ie for *I. elegans*, and Ig for *I. graellsii*. For each population, the sampling year (Year), Ecology (sympatric or allopatric), number of individual samples (*N*), number of alleles observed across all loci (Alleles#), the observed heterozygosity (*H*_O_), and expected heterozygosity (*H*_e_) are given

Population		Species	Year	Ecology	*N*	Alleles#	*H*_o_	*H*_e_
Allopatric populations								
Kaiserslautern	Germany	Ie	2009	Allopatric	18	100	0.72	0.76
Gran Sasso	Italy	Ie	2008	Allopatric	19	97	0.57	0.71
Liverpool	UK	Ie	2008	Allopatric	15	76	0.65	0.70
Córdoba	Spain	Ig	2008	Allopatric	13	43	0.68	0.52
Ribeira de Cobres	Portugal	Ig	2008	Allopatric	16	52	0.63	0.55
Parapatric populations								
Vigueirat	France	Ie	2008	Allopatric	17	86	0.62	0.71
Menorca	Spain	Ie	2008	Allopatric	7	32	0.33	0.40
Introgressed populations								
Doniños	Spain	Ie	2007	Sympatry (introgressed)	20	79	0.63	0.68
Arreo	Spain	Ie	2008	Sympatry (introgressed)	17	112	0.67	0.74
Baldajo	Spain	Ie	2008	Sympatry (introgressed)	19	96	0.57	0.74
Alfaro	Spain	Ie	2008	Sympatry (introgressed)	20	105	0.64	0.73
Europa	Spain	Ie	2008	Sympatry (introgressed)	20	90	0.68	0.75
Amposta	Spain	Ie	2008	Sympatry (introgressed)	20	107	0.66	0.74
Marjal del Moro	Spain	Ie	2008	Sympatry (introgressed)	20	93	0.68	0.72

### 2.4 Microsatellite data analyses

Twelve microsatellites were used to assess the population level of genetic diversity in terms of allele frequencies, observed heterozygosity (*H*_O_), and allelic richness in the program GenAlEx 6.503 (Peakall & Smouse, 2012). Pairwise population differentiation was assessed by calculating *F*_ST_ (Weir & Cockerham, 1984) and Dest (Jost, 2008) in the R v3.1.2 (R Development Core Team 2016) package diveRsity (Keenan, McGinnity, Cross, Crozier, & Prodöhl, 2013) using 1,000 bootstraps for the calculation of 95% confidence intervals. structure version 2.2.3 (Pritchard, Stephens, & Donnelly, 2000) was used to quantify admixture proportions of each population for F_1_‐hybrids and backcrosses (Beaumont et al., 2001; Hansen & Mensberg, 2009; Sanz, Araguas, Fernández, Vera, & García‐Marín, 2009; Vähä & Primmer, 2006). We conducted admixture analyses in structure to assign *Ischnura* individuals (*N* = 156) from the parapatric (Vigueirat and Menorca) and sympatric populations (Doniños, Arreo, Baldajo, Alfaro, Estanyo de Europa, Amposta, and Marjal del Moro) into each of two genetic clusters, one representing *I. graellsii* genotypes and one *I. elegans*. We used the “prior population information” option to facilitate the clustering process of the reference individuals (i.e., allopatric *I. elegans*, and allopatric *I. graellsii*) and to calculate the admixture proportions (and ±90% credible regions) of each individual in parapatric and sympatric populations. This approach was used to measure the degree of introgression of *I. graellsii* genetic material into the genome of *I. elegans* in Spain. The model was run for *K *=* *2, where each cluster corresponded (to a very high extent) to *I. elegans* and *I. graellsii*, respectively. We used the “population flag” option to exclude parapatric and sympatric *I. elegans* as reference individuals, which implied that the clustering process was based on only *I. graellsii* samples and *I. elegans* samples collected outside of Spain (i.e., outside the hybrid zone). The model was run for 100,000 MCMC replicates, after an initial burn‐in period of 50,000 replicates, using the admixture model and correlated frequencies for one iteration.

Simulated hybrid genotypes were generated to compare them with the levels of admixture detected in natural populations. That comparison will allow us to confirm the pattern of hybridization in each population. To generate simulated genotypes of hybrids and backcrosses, we applied the program Hybrid‐Lab (Nielsen, Bach, & Kotlicki, 2006) using the genotypes of 29 individuals of *I. graellsii* and 52 genotypes of *I. elegans* collected outside of Spain as initial genotypes. We generated 50 genotypes of each of the following crosses: first‐generation hybrid (F_1_; i.e., *I. graellsii* × *I. elegans*), first backcross with *I. elegans* (1EB; i.e., F_1_ × *I. elegans*), first backcross with *I. graellsii* (1GB; F_1_ × *I. graellsii*), second backcross with *I. elegans* (2EB; 1EB × *I. elegans*), third backcross with *I. elegans* (3EB; 2EB × *I. elegans*), and forth backcross with *I. elegans* (4EB; 3EB × *I. elegans*). We then evaluated the admixture proportions (±90% credible intervals) of these artificial crosses with STRUCTURE. To determine the level of introgression of *I. graellsii* into the Spanish *I. elegans* populations, the individual admixture proportions of the *I. elegans* samples from Spain were compared with the admixture proportion for the artificial hybrids and backcrosses.

To evaluate whether ongoing admixture in the sympatric area has resulted in genomewide linkage disequilibrium, we performed genotypic linkage disequilibrium analyses between each pair of loci within each of the following three regions: allopatric *I. elegans* populations (*n* = 5 populations), sympatric populations (*n* = 7), and allopatric *I. graellsii* populations (*n* = 2) using Genepop (web‐version, option 2, test for each pair of loci in each region using log likelihood tests with the following Markov chain parameter settings: dememorization number: 1,000; number of batches: 100; number of iterations per batch: 1,000).

### 2.5 Niche analyses

To evaluate whether allopatric and sympatric *I. elegans* and *I. graellsii* share the same fundamental niche, we generated individual niche models for *I. elegans* allopatric (712 presence points), *I. elegans* sympatric (265 presence points), *I. graellsii* allopatric (649 presence points), *I. graellsii* sympatric (376 presence points), and all presences of *I. graellsii* combined (1,025 presence points). These presence data points were obtained from a much larger data set that was generated for a previous study on all European *Ischnura* species. Species niches were modeled with the MAXENT 3.3.3k algorithm (Phillips, Anderson, & Schapire, 2006) using bioclimatic variables derived from the WorldClim 1.4 (http://www.worldclim.org) data set (Hijmans, Cameron, Parra, Jones, & Jarvis, 2005) at a 1 × 1 km cell size. To establish a set of uncorrelated climatic variables, we intersected the variables with 10,000 random points selected in the extension of the study area, ran an exploratory data analysis and a correlation analysis, and subsequently eliminated one of the variables in each pair with a Pearson correlation value >0.7. The final data set included three variables: Annual Mean Temperature (bio_1), Temperature Annual Range (bio_7), and Annual Precipitation (bio_12). MAXENT models were constructed setting most parameters to default (“Auto features,” convergence = 10^−5^, maximum number of iterations = 500, background = 10,000 points) and varying the prevalence (0.5, 0.6, and 0.7) and the regularization value β (1, 2, and 3) to find which combination generated the best outcomes (estimated using the highest area under the curve, or AUC value) while minimizing the number of model parameters, as well as producing “closed,” bell‐shaped response curves.

We subsequently performed niche identity and background similarity tests, comparing the actual ecological niche models with random models generated from 100 pseudoreplicate data sets (Warren, Glor, & Turelli, 2008) using Schoener's *D* and a modified Hellinger distance's *I* statistics as implemented in the package “phyloclim” (Heibl & Calenge, 2014) for R v3.1.2 (R Development Core Team 2016). The values of the two statistics vary between 0 (no niche overlap) and 1 (identical ecological niche). The background similarity test is particularly pertinent in this study because we seek to test whether the niches from the two allopatric populations are more different than would be expected given the underlying environmental differences between the regions in which they occur.

## 3 RESULTS

### 3.1 Molecular population structure

Microsatellite analyses showed that the populations exhibited a high degree of genetic variation, as shown by the pronounced genetic diversity at each locus (Table 1). The total number of alleles over all loci per population ranged between 32 (Menorca) and 112 (Arreo). Estimates of observed heterozygosity (*H*_O_) ranged between 0.33 and 0.72 and the expected heterozygosity (*H*_E_) ranged between 0.40 and 0.76 (Table 1). Genetic diversity measures among the *I. elegans* populations were slightly higher in the introgressed populations compared with the allopatric ones, but this was not significant (introgressed populations, *n* = 7: mean *H*_O_ = 0.65, mean *H*_E_ = 0.73; allopatric populations, *n* = 5: mean *H*_O_ = 0.58, mean *H*_E_ = 0.66, *p* = .2). The number of alleles was also higher in introgressed populations, with a mean number of 97, compared with a mean number of 78 in the allopatric populations.

The pairwise population differentiation measures *F*_ST_ and Dest ranged between <0.01–0.14, and <0.01–0.41, among *I. elegans* populations, respectively. When including the two populations of *I. graellsii* in this comparison, the upper *F*_ST_ value range increased to 0.25 and the upper Dest value range to 0.63. Overall, the pairwise population differentiation patterns across the range are heterogeneous, but some spatial patterns emerge. In comparison with the allopatric *I. elegans* populations, the introgressed populations are generally more similar to the *I. graellsii* populations (Table 2). This is consistent with the idea that this zone is characterized by ongoing introgression between the species. Further, populations close to the Mediterranean appear to be more closely related to *I. graellsii* compared with populations from central Spain (Table 2). Of all introgressed populations, Doniños has the highest *F*_ST_ and Dest values when compared to the allopatric *I. elegans* populations, in line with its geographic location (it is the most isolated population in northwest Spain, Sánchez‐Guillén, Wellenreuther, et al., 2011). This finding is also consistent with the hypothesis that this location was founded by immigrants from different regions than central and eastern populations (Sánchez‐Guillén, Wellenreuther, et al., 2011; Wellenreuther et al., 2011). Lastly, the allopatric population on the small island Menorca showed *F*_ST_ and Dest values with other *I. elegans* populations similar to those with *I. graellsii*. This could be the result of strong genetic drift on a small island, possibly associated with an initially small founding population. The overall genetic population differentiation showed a clear geographic signal, as indicated by the correlation between the spatial distance between populations and their *F*_ST_ values (*r* = .367, one‐tailed Mantel test *p* < .001). Population comparisons with Menorca appeared as clear outliers in this regression, however. We therefore run a second isolation by distance analysis after removing Menorca and found that the spatial signal was even stronger after the removal (*r* = .568, one‐sided Mantel test *p* < .001).

**Table 2:**
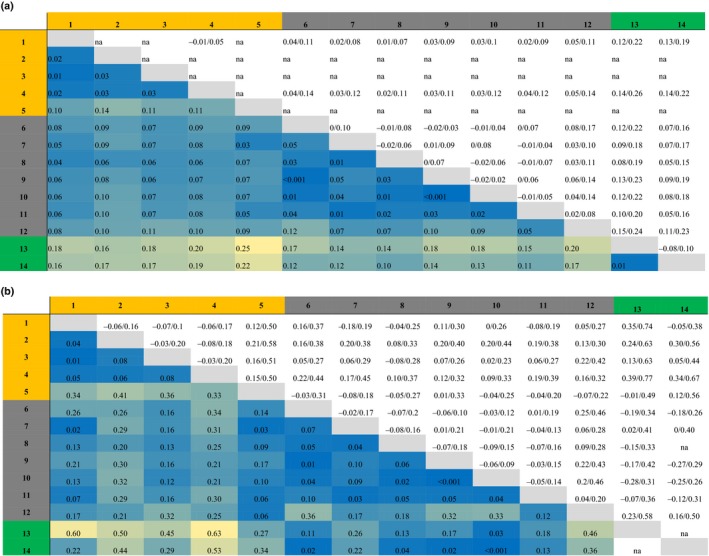
Differentiation between *I. elegans* populations with Weir and Cockerham's *F*_ST_ shown in (A) and Jost's DEST in (B) in the lower diagonal with the blue and yellow shading indicating the *F*_ST_ and DEST values (high values are yellow shaded, and low values have dark blue shading). The upper diagonal shows the ±95% confidence intervals. Populations are denoted by numbers (1 = Kaiserslautern, 2 = Gran Sasso, 3 = Liverpool, 4 = Vigueirat, 5 = Menorca, 6 = Doniños, 7 = Arreo, 8 = Baldajo, 9 = Alfaro, 10 = Estanyo de Europa, 11 = Amposta and 12 = Marjal del Moro, 13 = Córdoba and 14 = Ribeira de Cobres). Populations are colored according to their ecology, with all allopatric *I. elegans* populations in orange, the introgressed *I. elegans* populations in dark gray, and the two *I. graellsii* populations in green

**Figure 2:**
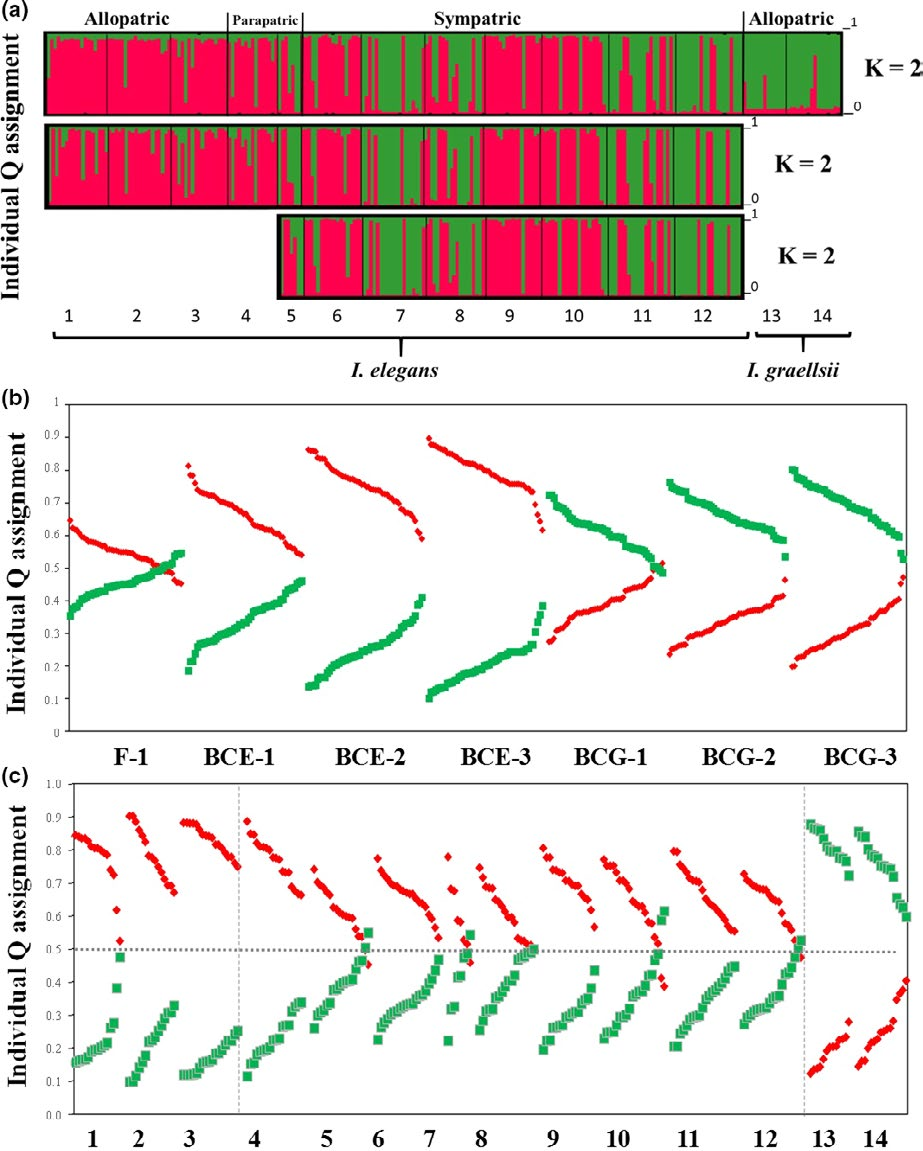
Admixture analysis in structure. Panel A shows the estimated admixture proportion of each individual (±90% credible intervals) to the *Ischnura elegans* cluster. Each individual is represented by a single vertical line broken into two segments which are proportional to the estimated membership to each of the two genetic clusters [Q_1_ for *I. elegans* (black), and Q_2_ for *Ischnura graellsii* (gray)]. The A panel represents 237 genotypes of three allopatric (Liverpool, Kaiserslautern, and Gran Sasso, respectively), two parapatric (Vigueirat and Menorca), seven sympatric *I. elegans* populations (Estanyo de Europa, Amposta, Marjal del Moro, Arreo, Alfaro, Doniños, and Baldajo), and two allopatric *I. graellsii* populations (Ribeira de Cobres and Córdoba). Panel B represents admixture proportions for the 350 artificial hybrids generated with the program hybrid‐lab [first‐generation hybrid (F_1_; i.e., *I. graellsii* × *I. elegans*), first backcross with *I. elegans* (1EB; i.e., F_1_ × *I. elegans*), first backcross with *I. graellsii* (1GB; F_1_ × *I. graellsii*), second backcross with *I. elegans* (2EB; 1EB × *I. elegans*), third backcross with *I. elegans* (3EB; 2EB × *I. elegans*), and forth backcross with *I. elegans* (4EB; 3EB × *I. elegans*)]. Panel C represents individual admixture proportions (±90% credible intervals) of two parapatric populations, seven sympatric *I. elegans* populations, followed by the three allopatric *I. elegans* populations, and the two allopatric *I. graellsii* populations

When investigating population admixture with STRUCTURE using the Δ*K*‐method, two clusters were suggested as most likely, one mainly corresponding to allopatric *I. elegans* (European populations outside Spain) and one mainly to allopatric *I. graellsii* populations (Table 2, Figure 2a). The introgressed populations were classified as a mixture of these two clusters (Table 3, Figure 2a). Most individuals of the European *I. elegans* outside Spain (72%) and *I. graellsii* (72%) were assigned with a certainty of at least 75% to each of these clusters. However, only 54% of the parapatric and 8% of the *I. elegans* of the sympatric populations were assigned with a certainty of at least 75% to the *I. elegans* cluster (Table 3; Figure 2a). The rest of the sympatric *I. elegans* were intermediate between the two clusters, suggesting a significant degree of introgressed *I. graellsii* alleles (Table 3; Figure 2a).

**Table 3:** Summary of the results from the *K* = 2 admixture models in structure for *I. elegans* and *I. graellsii* showing the number of individuals per population assigned to different admixture proportion categories (based on individual assignment to the red cluster in Figure 2)

Population origin	*N*	Species	≥75	(74–56)	(55–50)	(49–26)	≤25
Allopatric populations							
Germany, Italy and UK	52	Ie	38	14			
Spain and Portugal	29	Ig				8	21
Parapatric populations							
Vigueirat	16	Ie	12	3	1		
Menorca	8	Ie	1	4	2	1	
Introgressed populations							
Doniños	20	Ie	3	17			
Arreo	17	Ie	3	14			
Baldajo	19	Ie	1	15	1	2	
Alfaro	20	Ie	4	12	2	2	
Estanyo de Europa	18	Ie		14	2	2	
Amposta	20	Ie	1	18	1		
Marjal del Moro	18	Ie		12	6		

The admixture proportions of the artificial hybrids and backcrosses ranged between 19 and 89% (Table 4; Figure 2b). The *F*_1_ showed admixture proportions to *I. elegans* between 45% and 68%; the three *I. elegans* backcrosses (1‐3EB) between 54% and 89%, and the three *I. graellsii* backcrosses (1‐3GB) between 19% and 61% (Table 4; Figure 2b). Three conservative assignment groups were defined based on the results from the artificial hybrids: F_1_ and BCE‐1 and BCG‐1 ranging between 55% and 50%; backcrosses with *I. elegans* ≥75; and backcrosses (only BCG‐2 and BCG‐3) with *I. graellsii* <25%. The parapatric and sympatric *I. elegans* genotypes (Table 3; Figure 2c) were assigned to F_1_, BCE‐1, and BCG‐1 (*N* = 25 individuals), backcrosses with *I. elegans* (*N* = 124) and *I. graellsii* (*N* = 7).

**Table 4:** Summary of the results from the admixture models in STRUCTURE for artificial hybrids and backcrosses: first‐generation hybrid (F1; i.e., *I. graellsii* × *I. elegans*), first backcross with *I. elegans* (1EB; i.e., F1 × *I. elegans*), first backcross with *I. graellsii* (1GB; F1 × *I. graellsii*), second backcross with *I. elegans* (2EB; 1EB × *I. elegans*), third backcross with *I. elegans* (3EB; 2EB × *I. elegans*)

Type of crosses	*N*	≥75	(74–56)	(55–50)	(49–26)	≤25
F1	50	0	17	24	9	0
1GB	50	0	0	3	47	0
2GB	50	0	0	0	49	1
3GB	50	0	0	0	40	10
1EB	50	4	42	4	0	0
2EB	50	27	23	0	0	0
3EB	50	42	8	0	0	0

The genotypic linkage disequilibrium analyses between pairs of loci showed significant associations for allopatric *I. elegans* populations. Of the 55 pairs of loci that could be tested, four deviated significantly from equilibrium (Table S1). Furthermore, when testing the sympatric *I. elegans* populations 10 of the 66 pairs of loci deviated significantly from equilibrium (Table S1). In contrast, no significant deviations from equilibrium were detected for allopatric *I. graellsii* populations (21 pairs of loci were tested) (Table S1).

### 3.2 Niche analyses

We tested if the environmental niche models of the two species were more different than expected by chance (identity test), and found that all pairwise comparisons of sympatric and allopatric populations of *I. elegans* and *I. graellsii* were less similar than expected by chance (Table 5). This indicates that all populations (*I. elegans* sympatric and allopatric and *I. graellsii* sympatric and allopatric) are found to occupy different niches, despite often occurring in close geographic proximity.

**Table 5:** Summary of niche identity and background similarity tests. “Divergence” indicates that the compared populations show significant divergence (overlap is less than expected), while “Conservatism” indicates niche conservatism (overlap values are more similar than expected). “**” indicates a significant (*p *< .01?), and “n.s.” no significant, difference between expected and observed overlap

*a*	*b*	Schoener's *D*	Modified Hellinger *I*	Niche D/I
Niche identity test				
*I. elegans* allopatric	*I. elegans* sympatric	0.398**	0.679**	Not identical/not identical
*I. graellsii* allopatric	*I. graellsii* sympatric	0.508**	0.793**	Not identical/not identical
*I. elegans* allopatric	*I. graellsii* allopatric	0.291**	0.564**	Not identical/not identical
*I. elegans* allopatric	*I. graellsii* sympatric	0.499**	0.767**	Not identical/not identical
*I. elegans* sympatric	*I. graellsii* allopatric	0.593**	0.844**	Not identical/not identical
*I. elegans* sympatric	*I. graellsii* sympatric	0.733**	0.935**	Not identical/not identical

*a*	*b*	Schoener's *D*		Modified Hellinger *I*		Niche D/I	
		*a* → *b*	*b* → *a*	*a* → *b*	*b* → *a*	*a* → *b*	*b* → *a*
Background similarity test							
*I. elegans* allopatric	*I. elegans* sympatric	0.392**	0.672**	0.392**	0.672**	Divergence/divergence	Divergence/divergence
*I. graellsii* allopatric	*I. graellsii* sympatric	0.511*	n.s.	n.s.	n.s.	Conservatism/similar	Similar/similar
*I. elegans* allopatric	*I. graellsii* allopatric	0.295**	0.567**	0.295**	0.567**	Divergence/divergence	Divergence/divergence
*I. elegans* allopatric	*I. graellsii* sympatric	n.s.	n.s.	n.s.	n.s.	Similar/similar	Similar/similar
*I. elegans* sympatric	*I. graellsii* allopatric	0.589**	0.842**	0.589**	0.842**	Conservatism/conservatism	Conservatism/conservatism
*I. elegans* sympatric	*I. graellsii* sympatric	0.735**	0.937**	0.735**	0.937**	Conservatism/conservatism	Conservatism/conservatism

**Figure 3:**
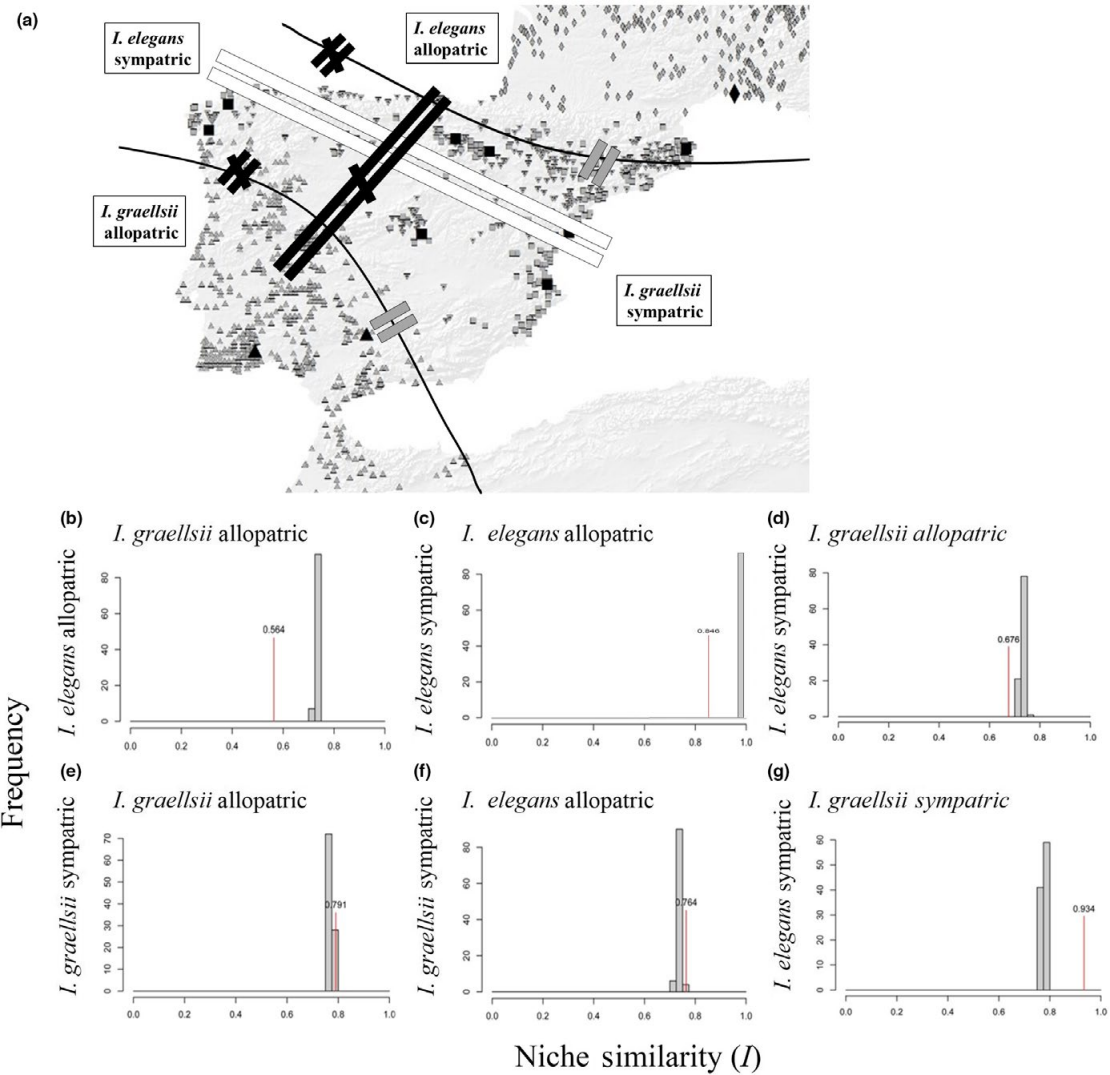
Niche analyses. Results of background similarity test, showing modified Hellinger distance *I*. The observed similarity between niches is indicated with the red lines with Hellinger values on top, while histograms indicate the null distribution of ecological niche distance generated randomly. Hellinger distance *I* ranges from 0 (complete different) to 1 (identical). (a) Map of compared region niches (*I. elegans* from allopatry, *I. elegans* from sympatry, *I. graellsii* from allopatry, and *I. graellsii* from sympatry). Black unequal symbol indicates that niches are more different that by change; gray equal symbols indicate no differences between niches, and white equal symbol indicates that niches are more equal than by change. (b–d) The observed similarities were lower than their respective null distribution for random niche models. (e and f) The observed similarities were similar that their respective null distribution for random niche models. (g) The observed similarities were higher than their respective null distribution for random niche models

We also carried out background niche tests to determine whether the species’ ranges were more different from one another than expected based on the environmental background differences (Figure 3a). Our background niche analyses using Hellinger's *I* demonstrated that both species from their allopatric distributions show decreased niche similarity (Figure 3b). Interestingly, *I. elegans* in the sympatric portion shows decreased niche similarity with *I. elegans* in the allopatric portion (Figure 3c) and *I. graellsii* in the allopatric portion of their range (Figure 3d) as both similarity scores were lower than the generated null hypotheses, while *I. graellsii* from the sympatric portion was not statistically lower or higher (than what would be expected by random) compared with *I. graellsii* from allopatry (Figure 3e) or *I. elegans* from allopatry (Figure 3f). Our background niche analyses also demonstrated that *I. elegans* in the sympatric portion showed increased niche similarity with *I. graellsii* in the sympatric portion of their range (Figure 3g), as indicated by the similarity score (red line) being higher than the null hypothesis of niche equivalency. Together this indicates that it has been a niche displacement in *I. elegans* resulting in that sympatric populations of *I. elegans* now share the same niche space as *I. graellsii*, but not anymore with *I. elegans* from allopatry. However, we found no evidence for niche displacement in the sympatric *I. graellsii* populations, which still share a niche with the allopatric *I. graellsii* populations.

## 4 DISCUSSION

It is recognized that hybrid zones vary greatly in their structures depending on the degree of the molecular and ecological differentiation between the introgressing species and affect associated levels of dispersal (Buggs, 2007; Glotzbecker, Walters, & Blum, 2016). Our molecular results suggest that introgressive hybridization between *I. elegans* and *I. graellsii* is chronic, and our niche modeling indicates that this introgression is enabling the niche shift of introgressed *I. elegans* into the Iberian Peninsula. Our genetic data corroborates previous findings of enhanced genetic diversity of *I. elegans* populations inhabiting the hybrid zone, supporting the view that hybridization can be a source of new adaptive variation for a species (Sánchez‐Guillén, Hansson, et al., 2011). Admixture results were in line with this finding, grouping *I. elegans* and *I. graellsii* in independent clusters, with the sympatric populations in the hybrid zone as an intermediate cluster between the two. Specifically, only a small proportion of parapatric and sympatric *I. elegans* populations could be assigned to either of the two clusters, with the majority falling in intermediate clusters, indicating a significant degree of introgressed *I. graellsii* alleles; a finding that was also supported by the admixture proportions of the artificial hybrids.

The niche modeling further supported the idea that *I. elegans* has shifted its environmental niche following hybridization and genetic introgression with *I. graellsii*. Niche use of allopatric *I. elegans* (temperate and cold regions) was clearly different from *I. graellsii* (temperate and warm regions), whereas sympatric and introgressed *I. elegans* were currently expanding its distribution to inhabit the same niche space as its sister species, *I. graellsii* into the dry Mediterranean regions (center Spain). Our niche similarity results thus suggest that introgressive hybridization may be enabling *I. elegans* to inhabit the center of Spain, a region that was previously only occupied by *I. graellsii* (Sánchez‐Guillén, Wellenreuther, et al., 2011). Support for this comes from the finding that *I. elegans* populations in southern Europe have seldom been able to colonize similar hot and dry habitats, although the general conditions of the niches are quite similar to Corsica or Sardinia, where *I. genei* is the dominant species (Askew, 2004), but we have already found the first evidence of hybridization between *I. elegans* and *I. genei* (Sánchez‐Guillén, Córdoba‐Aguilar, Cordero‐Rivera, & Wellenreuther, 2014). Colonization of the Iberian Peninsula by *I. elegans* was first achieved by colonizing the northernmost areas close to the Pyrenees, an area that falls inside *I. elegans*’ niche breadth (Sánchez‐Guillén, Wellenreuther, et al., 2011). However, it appears that over time, and thus, with ongoing introgression, *I. elegans* has been able to expand its range rapidly into the south‐western areas that are characterized by a much humid and warm climate. Our results thus indicate that *I. elegans* population in the Iberian Peninsula have undergone a niche shift, likely because the backcross with *I. elegans* offspring showed increased competitive fitness. This introgressed *I. elegans* on the Iberian Peninsula may indicate the start of a new evolutionary lineage that could over time and with ongoing divergence evolve into a reproductively isolated species. Alternatively, genetic swamping could replace rare lineages from some places across the hybrid zone, or by demographic swamping, where population growth rates are reduced due to the wasteful production of maladaptive hybrids (Todesco et al., 2016).

Adaptation to local environments can be achieved by selection on standing genetic variation (i.e., variation present in the species at the time of the environmental change) or de novo mutations. Another less explored path for species to acquire beneficial alleles is through introgression via interspecific hybridization (Arnold, 1997; Glotzbecker et al., 2016; Rieseberg & Wendel, 1993; Whitney, Randell, & Rieseberg, 2006). The outcomes of such introgressive hybridization are manifold and include the fusion of species, genetic swamping of one species by another, elicit reinforcement of reproductive isolation between incompletely isolated species, transfer of genetic material between species, potentially facilitating their adaptive evolution, and ultimately, the origin of new species (Seehausen, 2004; Zemanova, Knop, & Heckel, 2017). Our data suggest that the introgressive hybridization between *I. elegans* and *I. graellsii* is potentially providing a new source of alleles for the populations in the hybrid zone and that this may enable their contemporary and possibly also future spread. Introgression can also happen without an obvious impact on the genetic diversity if the recombinant genotypes are retained in a narrow contact zone between two species via a balance between dispersal into the contact zone and selection against hybrids. However, in the current case, it seems that the hybrid zone is dynamic and moving, and this means that the genetic impact will not be confined to a small geographic area. This indicates that the genetic exchange between *I. elegans* and *I. graellsii* may have widespread and prolonged consequences for these *Ischnura* species in southern Europe, and that the final impact of the introgressive hybridization on the species diversity and maintenance is difficult to predict with certainty.

The view of prolonged hybridization in the evolution of biological diversity has changed over time. Some thought of hybrids as the raw materials of evolution and a creative source of functional novelty (Buggs, 2007; Rieseberg & Wendel, 1993). Others, however, thought that hybridization presented an evolutionary dead end (Mayr, 1963). These contrasting views were based on the fact that at least 25% of plant species, but only 10% of animal species, mostly phylogenetically recent species, are involved in hybridization and potential introgression with other species (Mallet, 2005). Consequently, many plant studies viewed hybridization as an important source of new genetic variation and a frequent component of the evolutionary history of many plant species (Harrison, 1993), whereas hybridization within the animal kingdom was viewed as a rare occurrence (Arnold & Hodges, 1995). Early work (Anderson, 1949; Anderson & Stebbins, 1954; Lewontin & Birch, 1966) emphasized the importance of reshuffling segregating genetic variation via hybridization. Since then, some empirical studies have emerged in support of this view, and these indicate that hybridization provides an important source of genetic variation on which selection can act and that its adaptive role is even more widespread, among both plants and animals, than previously believed (Dowling & Secor, 1997; Hedrick, 2013; Rieseberg & Wendel, 1993; Rius & Darling, 2014). Awareness is also increasing that this mixing of gene pools can in some cases lead to the potential loss or displacement of genotypically distinct species, something which can be especially problematic for rare organisms coming into contact with more abundant ones (Zemanova et al., 2017). Our study adds a new empirical example from a nonmodel animal species to this idea and shows that genetic introgression may be facilitating the range expansion of some hybridizing species via niche expansion.

In conclusion, the molecular data and the niche modeling together suggest adaptive trait introgression from the native *I. graellsii* into the expanding *I. elegans*, and that this is enabling the niche shift of introgressed *I. elegans* (sensu Arnold, 2004). A recent revision of the distribution of both species further showed that in eastern Spain, *I. graellsii* has almost been completely replaced by *I. elegans,* and thus, understanding the expansion process is important for conservation (Sánchez‐Guillén, Muñoz, Rodríguez‐Tapia, Arroyo, & Córdoba‐Aguilar, 2013; Sánchez‐Guillén, Wellenreuther, et al., 2011). Indeed, the colonization process in the north‐western, north‐central, and eastern region has been affected by an asymmetric hybridization process (Sánchez‐Guillén et al., 2012), resulting in the introgression of genes from *I. graellsii* into *I. elegans*. The reported adaptive introgression may thus play a role in increasing *I. elegans* ability to respond to a changing climate. The ongoing interspecific gene flow with *I. graellsii* may increase the limited adaptive potential that results from standing genetic variation and mutation alone, enabling a quicker demographic recovery in response to changing environments. Taken together, our results corroborate the view that hybridization can play an important and creative role in adaptive evolution.

## CONFLICT OF INTEREST

None declared.

## AUTHOR CONTRIBUTIONS

MW, AC‐R, and RAS‐G designed research. RAS‐G, AC‐R, and JRC‐R collected field samples. RAS‐G, JRMW, BH, JM, and RAS‐G analyzed data. All authors contributed to writing the manuscript.

## References

[ece34024-bib-0001] Anderson, E. (1949). Introgressive hybridization. London, UK: Chapman & Hall https://doi.org/10.5962/bhl.title.4553

[ece34024-bib-0002] Anderson, E. , & Stebbins, G. (1954). Hybridization as an evolutionary stimulus. Evolution, 8, 378–388. https://doi.org/10.1111/j.1558-5646.1954.tb01504.x

[ece34024-bib-0003] Arnold, M. L. (1997). Natural hybridization and evolution. New York, NY: Oxford University Press.

[ece34024-bib-0004] Arnold, M. L. (2004). Transfer and origin of adaptations through natural hybridization: Were Anderson and Stebbins right? The Plant Cell, 16, 562–570. https://doi.org/10.1105/tpc.160370 15004269 10.1105/tpc.HistPersp PMC540259

[ece34024-bib-0005] Arnold, M. L. , & Hodges, S. A. (1995). Are natural hybrids fit or unfit relative to their parents? Trends in Ecology & Evolution, 10, 67–71. https://doi.org/10.1016/S0169-5347(00)88979-X 21236955 10.1016/S0169-5347(00)88979-X

[ece34024-bib-0006] Askew, R. R. (2004). The dragonflies of Europe. Colchester, UK: B H & A Harley Ltd.

[ece34024-bib-0007] Baack, E. J. , & Rieseberg, L. H. (2007). A genomic view of introgression and hybrid speciation. Current Opinion in Genetics & Development, 17, 513–518. https://doi.org/10.1016/j.gde.2007.09.001 17933508 10.1016/j.gde.2007.09.001 PMC2173880

[ece34024-bib-0008] Beaumont, M. , Barratt, E. M. , Gottelli, D. , Kitchener, A. C. , Daniels, M. J. , Pritchard, J. K. , & Bruford, M. W. (2001). Genetic diversity and introgression in the Scottish wildcat. Molecular Ecology, 10, 319–336. https://doi.org/10.1046/j.1365-294x.2001.01196.x 11298948 10.1046/j.1365-294x.2001.01196.x

[ece34024-bib-0009] Brand, C. L. , Kingan, S. B. , Wu, L. , & Garrigan, D. (2013). A selective sweep across species boundaries in Drosophila. Molecular Biology and Evolution, 30, 2177–2186. https://doi.org/10.1093/molbev/mst123 23827876 10.1093/molbev/mst123 PMC3748358

[ece34024-bib-0010] Buggs, R. (2007). Empirical study of hybrid zone movement. Heredity, 99, 301 https://doi.org/10.1038/sj.hdy.6800997 17611495 10.1038/sj.hdy.6800997

[ece34024-bib-0011] Canestrelli, D. , Porretta, D. , Lowe, W. H. , Bisconti, R. , Carere, C. , & Nascetti, G. (2016). The tangled evolutionary legacies of range expansion and hybridization. Trends in Ecology & Evolution, 31, 677–688. https://doi.org/10.1016/j.tree.2016.06.010 27450753 10.1016/j.tree.2016.06.010

[ece34024-bib-0012] Chauhan, P. , Hansson, B. , Kraaijeveld, K. , de Knijff, P. , Svensson, E. I. , & Wellenreuther, M. (2014). De novo transcriptome of *Ischnura elegans* provides insights into sensory biology, colour and vision genes. BMC Genomics, 15, 808 https://doi.org/10.1186/1471-2164-15-808 25245033 10.1186/1471-2164-15-808 PMC4182773

[ece34024-bib-0013] Chauhan, P. , Wellenreuther, M. , & Hansson, B. (2016). Transcriptome profiling in the damselfly *Ischnura elegans* identifies genes with sex‐biased expression. BMC Genomics, 17, 985 https://doi.org/10.1186/s12864-016-3334-6 27905879 10.1186/s12864-016-3334-6 PMC5131402

[ece34024-bib-0014] Cordero, A. , & Andrés, J. A. (1996). Colour polymorphism in Odonates: Females that mimic males? Journal of the British Dragonfly Society, 12, 50–60.

[ece34024-bib-0015] Coyne, J. A. , & Orr, H. A. (2004). Speciation. Sunderland, MA: Sinauer Associates.

[ece34024-bib-0016] Dowling, T. E. , & Secor, C. L. (1997). The role of hybridisation and introgression in the diversification of animals. Annual Review of Ecology, Evolution, and Systematics, 28, 593–619. https://doi.org/10.1146/annurev.ecolsys.28.1.593

[ece34024-bib-0017] Glotzbecker, G. J. , Walters, D. M. , & Blum, M. J. (2016). Rapid movement and instability of an invasive hybrid swarm. Evolutionary Applications, 9, 741–755. https://doi.org/10.1111/eva.12371 27330551 10.1111/eva.12371 PMC4908461

[ece34024-bib-0018] Gómez, J. M. , González‐Megías, A. , Lorite, J. , Abdelaziz, M. , & Perfectti, F. (2015). The silent extinction: Climate change and the potential hybridization‐mediated extinction of endemic high‐mountain plants. Biodiversity and Conservation, 24, 1843–1857. https://doi.org/10.1007/s10531-015-0909-5

[ece34024-bib-0019] Hansen, M. M. , & Mensberg, K.‐L. D. (2009). Admixture analysis of stocked brown trout populations using mapped microsatellite DNA markers: Indigenous trout persist in introgressed populations. Biology Letters, 5, 656–659. https://doi.org/10.1098/rsbl.2009.0214 19515653 10.1098/rsbl.2009.0214 PMC2781948

[ece34024-bib-0020] Harrison, R. (1993). Hybrid zones and the evolutionary process. New York, NY: Oxford University Press.

[ece34024-bib-0021] Hedrick, P. W. (2013). Adaptive introgression in animals: Examples and comparison to new mutation and standing variation as sources of adaptive variation. Molecular Ecology, 22, 4606–4618. https://doi.org/10.1111/mec.12415 23906376 10.1111/mec.12415

[ece34024-bib-0022] Heibl, C. , & Calenge, C. (2014). phyloclim: Integrating phylogenetics and climatic niche modelling. R package version 0.0. 1.

[ece34024-bib-0023] Hijmans, R. J. , Cameron, S. E. , Parra, J. L. , Jones, P. G. , & Jarvis, A. (2005). Very high resolution interpolated climate surfaces for global land areas. International Journal of Climatology, 25, 1965–1978. https://doi.org/10.1002/(ISSN)1097-0088

[ece34024-bib-0024] Huerta‐Sanchez, E. , Jin, X. , Asan, Z. , Bianba, B. M. , Peter, N. , Vinckenbosch, Y. , … Nielsen, R. (2014). Altitude adaptation in Tibetans caused by introgression of Denisovan‐like DNA. Nature, 512, 194–197. https://doi.org/10.1038/nature13408 25043035 10.1038/nature13408 PMC4134395

[ece34024-bib-0025] Jost, L. O. U. (2008). GST and its relatives do not measure differentiation. Molecular Ecology, 17, 4015–4026. https://doi.org/10.1111/j.1365-294X.2008.03887.x 19238703 10.1111/j.1365-294x.2008.03887.x

[ece34024-bib-0026] Kays, R. , Curtis, A. , & Kirchman, J. J. (2009). Rapid adaptive evolution of northeastern coyotes via hybridization with wolves. Biology Letters, 6(1):89‐93. http://doi.org/10.1098/rsbl.2009.0575. Epub 2009 Sep 23. 19776058 10.1098/rsbl.2009.0575 PMC2817252

[ece34024-bib-0027] Keenan, K. , McGinnity, P. , Cross, T. F. , Crozier, W. W. , & Prodöhl, P. A. (2013). diveRsity: An R package for the estimation and exploration of population genetics parameters and their associated errors. Methods in Ecology and Evolution, 4, 782–788. https://doi.org/10.1111/2041-210X.12067

[ece34024-bib-0028] Keller, I. , Wagner, C. , Greuter, L. , Mwaiko, S. , Selz, O. , Sivasundar, A. , … Seehausen, O. (2013). Population genomic signatures of divergent adaptation, gene flow and hybrid speciation in the rapid radiation of Lake Victoria cichlid fishes. Molecular Ecology, 22, 2848–2863. https://doi.org/10.1111/mec.12083 23121191 10.1111/mec.12083

[ece34024-bib-0029] Kelly, B. P. , Whiteley, A. , & Tallmon, D. (2010). The Arctic melting pot. Nature, 468, 891 https://doi.org/10.1038/468891a 21164461 10.1038/468891a

[ece34024-bib-0030] Lancaster, L. T. , Dudaniec, R. , Chauhan, P. , Wellenreuther, M. , Svensson, E. I. , & Hansson, B. (2016). Gene expression under thermal stress varies across a range‐expansion front. Molecular Ecology, 25, 1141–1156. https://doi.org/10.1111/mec.13548 26821170 10.1111/mec.13548

[ece34024-bib-0031] Lewontin, R. , & Birch, L. (1966). Hybridization as a source of variation for adaptation to new environments. Evolution, 20, 315–336. https://doi.org/10.1111/j.1558-5646.1966.tb03369.x 28562982 10.1111/j.1558-5646.1966.tb03369.x

[ece34024-bib-0032] Mallet, J. (2005). Hybridization as an invasion of the genome. Trends in Ecology & Evolution, 20, 229–237. https://doi.org/10.1016/j.tree.2005.02.010 16701374 10.1016/j.tree.2005.02.010

[ece34024-bib-0033] Mallet, J. , Wynne, I. R. , & Thomas, C. D. (2011). Hybridisation and climate change: Brown argus butterflies in Britain (Polyommatus subgenus Aricia). Insect Conservation and Diversity, 4, 192–199. https://doi.org/10.1111/j.1752-4598.2010.00122.x

[ece34024-bib-0034] Mayr, E. (1963). Animal species and evolution. Cambridge, MA: Belknap Press, Harvard University Press https://doi.org/10.4159/harvard.9780674865327

[ece34024-bib-0035] Monetti, L. , Sánchez‐Guillén, R. A. , & Cordero‐Rivera, A. (2002). Hybridization between *Ischnura graellsii* (Vander Linder) and *I. elegans* (Rambur) (Odonata: Coenagrionidae): Are they different species? Biological Journal of the Linnean Society, 76, 225–235. https://doi.org/10.1046/j.1095-8312.2002.00060.x

[ece34024-bib-0036] Nielsen, E. E. G. , Bach, L. A. , & Kotlicki, P. (2006). Hybridlab (version 1.0): A program for generating simulated hybrids from population samples. Molecular Ecology Notes, 6, 971–973. https://doi.org/10.1111/j.1471-8286.2006.01433.x

[ece34024-bib-0037] Ocharan Larrondo, F. J. (1987). Los odonatos de Asturias y de España: aspectos sistemáticos y faunísticos. PhD. Universidad de Oviedo, Oviedo.

[ece34024-bib-0038] Peakall, P. , & Smouse, R. (2012). GenAlEx 6.5: Genetic analysis in Excel. Population genetic software for teaching and research—an update. Bioinformatics, 28, 2537–2539. https://doi.org/10.1093/bioinformatics/bts460 22820204 10.1093/bioinformatics/bts460 PMC3463245

[ece34024-bib-0039] Phillips, S. J. , Anderson, R. P. , & Schapire, R. E. (2006). Maximum entropy modeling of species geographic distributions. Ecological Modelling, 190, 231–259. https://doi.org/10.1016/j.ecolmodel.2005.03.026

[ece34024-bib-0040] Pritchard, J. K. , Stephens, M. , & Donnelly, P. (2000). Inference of population structure using multilocus genotype data. Genetics, 155, 945–959. 10835412 10.1093/genetics/155.2.945 PMC1461096

[ece34024-bib-0041] R Development Core Team (2016). R: A language and environment for statistical computing. Vienna, Austria: R Foundation for Statistical Computing.

[ece34024-bib-0042] Rhymer, J. M. , & Simberloff, D. (1996). Extinction by hybridization and introgression. Annual Review of Ecology, Evolution, and Systematics, 27, 83–109. https://doi.org/10.1146/annurev.ecolsys.27.1.83

[ece34024-bib-0043] Rieseberg, L. H. (2009). Evolution: Replacing genes and traits through hybridization. Current Biology, 19, R119–R122. https://doi.org/10.1016/j.cub.2008.12.016 19211049 10.1016/j.cub.2008.12.016

[ece34024-bib-0044] Rieseberg, L. H. , & Brunsfeld, S. J. (2012). Plant introgression In Soltis P. S., Soltis D. E., & Doyle J. J. (Eds.), Molecular systematics of plants (p. 151). Boston, MA: Springer.

[ece34024-bib-0045] Rieseberg, L. H. , & Wendel, J. F. (1993). Introgression and its consequences in plants In Harrison R. G. (Ed.), Hybrid zones and the evolutionary process (pp. 70–109). New York, NY: Oxford University Press.

[ece34024-bib-0046] Rius, M. , & Darling, J. A. (2014). How important is intraspecific genetic admixture to the success of colonising populations? Trends in Ecology & Evolution, 29, 233–242. https://doi.org/10.1016/j.tree.2014.02.003 24636862 10.1016/j.tree.2014.02.003

[ece34024-bib-0047] Ryan, M. E. , Johnson, J. R. , & Fitzpatrick, B. M. (2009). Invasive hybrid tiger salamander genotypes impact native amphibians. Proceedings of the National Academy of Sciences of the United States of America, 106, 11166–11171. https://doi.org/10.1073/pnas.0902252106 19564601 10.1073/pnas.0902252106 PMC2703667

[ece34024-bib-0048] Sambrook, J. , Fritsch, E. F. , & Maniatis, T. (1989). Molecular cloning: A laboratory manual, 2nd ed. Cold Spring Harbor, NY: Cold Spring Harbor Laboratory Press.

[ece34024-bib-0049] Sánchez‐Guillén, R. , Cordero‐Rivera, A. , Rivas‐Torres, A. , Wellenreuther, M. , Bybee, S. , Hansson, B. , … Dumont, H. (under revision). The evolutionary history and adaptive significance of colour polymorphism in ischnuran damselflies.

[ece34024-bib-0050] Sánchez‐Guillén, R. A. , Córdoba‐Aguilar, A. , Cordero‐Rivera, A. , & Wellenreuther, M. (2014). Rapid evolution of prezygotic barriers in non‐territorial damselflies. Biological Journal of the Linnean Society, 113, 485–496. https://doi.org/10.1111/bij.12347

[ece34024-bib-0051] Sánchez‐Guillén, R. A. , Cordoba‐Aguilar, A. , Hansson, B. , Ott, J. , & Wellenreuther, M. (2016). Evolutionary consequences of climate‐induced range shifts in insects. Biological Reviews, 91, 1050–1064. https://doi.org/10.1111/brv.12204 26150047 10.1111/brv.12204

[ece34024-bib-0052] Sánchez‐Guillén, R. , Hansson, B. , Wellenreuther, M. , Svensson, E. , & Cordero‐Rivera, A. (2011). The influence of stochastic and selective forces in the population divergence of female colour polymorphism in damselflies of the genus *Ischnura* . Heredity, 107, 513–522. https://doi.org/10.1038/hdy.2011.36 21587304 10.1038/hdy.2011.36 PMC3242623

[ece34024-bib-0053] Sánchez‐Guillén, R. A. , Muñoz, J. , Rodríguez‐Tapia, G. , Arroyo, T. P. F. , & Córdoba‐Aguilar, A. (2013). Climate‐induced range shifts and possible hybridisation consequences in insects. PLoS ONE, 8, e80531 https://doi.org/10.1371/journal.pone.0080531 24260411 10.1371/journal.pone.0080531 PMC3829986

[ece34024-bib-0054] Sánchez‐Guillén, R. A. , Van Gossum, H. , & Cordero‐Rivera, A. (2005). Hybridization and the inheritance of female colour polymorphism in two Ischnurid damselflies (Odonata:Coenagrionidae). Biological Journal of the Linnean Society, 85, 471–481. https://doi.org/10.1111/j.1095-8312.2005.00506.x

[ece34024-bib-0055] Sánchez‐Guillén, R. A. , Wellenreuther, M. , Chávez‐Ríos, J. R. , Beatty, C. D. , Rivas‐Torres, A. , Velasquez‐Velez, M. , & Cordero‐Rivera, A. (2017). Alternative reproductive strategies and the maintenance of female color polymorphism in damselflies. Ecology and Evolution, 7, 5592–5602. https://doi.org/10.1002/ece3.3083 28811877 10.1002/ece3.3083 PMC5552903

[ece34024-bib-0056] Sánchez‐Guillén, R. A. , Wellenreuther, M. , & Cordero‐Rivera, A. S. (2012). Strong asymmetry in the relative strengths of prezygotic and postzygotic barriers between two damselfly sister species. Evolution, 66, 690–707. https://doi.org/10.1111/j.1558-5646.2011.01469.x 22380433 10.1111/j.1558-5646.2011.01469.x

[ece34024-bib-0057] Sánchez‐Guillén, R. A. , Wellenreuther, M. , Cordero‐Rivera, A. , & Hansson, B. (2011). Introgression and rapid species turnover in sympatric damselflies. BMC Evolutionary Biology, 11, 210 https://doi.org/10.1186/1471-2148-11-210 21767355 10.1186/1471-2148-11-210 PMC3146444

[ece34024-bib-0058] Sanz, N. , Araguas, R. M. , Fernández, R. , Vera, M. , & García‐Marín, J.‐L. (2009). Efficiency of markers and methods for detecting hybrids and introgression in stocked populations. Conservation Genetics, 10, 225–236. https://doi.org/10.1007/s10592-008-9550-0

[ece34024-bib-0059] Seehausen, O. (2004). Hybridization and adaptive radiation. Trends in Ecology & Evolution, 19, 198–207. https://doi.org/10.1016/j.tree.2004.01.003 16701254 10.1016/j.tree.2004.01.003

[ece34024-bib-0060] Svensson, E. , Abbott, J. , & Hardling, R. (2005). Female polymorphism, frequency dependence, and rapid evolutionary dynamics in natural populations. The American Naturalist, 165, 567–576. https://doi.org/10.1086/429278 10.1086/429278 15795853

[ece34024-bib-0061] Takahashi, Y. , Yoshimura, J. , Morita, S. , & Watanabe, M. (2010). Negative frequency‐dependent selection in female color polymorphism of a damselfly. Evolution, 64, 3620–3628. https://doi.org/10.1111/j.1558-5646.2010.01083.x 20629729 10.1111/j.1558-5646.2010.01083.x

[ece34024-bib-0062] Todesco, M. , Pascual, M. A. , Owens, G. L. , Ostevik, K. L. , Moyers, B. T. , Hübner, S. , … Rieseberg, L. H. (2016). Hybridization and extinction. Evolutionary Applications, 9, 892–908. https://doi.org/10.1111/eva.12367 27468307 10.1111/eva.12367 PMC4947151

[ece34024-bib-0063] Vähä, J.‐P. , & Primmer, C. R. (2006). The efficiency of model‐based Bayesian methods for detecting hybrid individuals under different hybridization scenarios and with different numbers of loci. Molecular Ecology, 15, 63–72. 16367830 10.1111/j.1365-294X.2005.02773.x

[ece34024-bib-0064] Warren, D. L. , Glor, R. E. , & Turelli, M. (2008). Environmental niche equivalency versus conservatism: Quantitative approaches to niche evolution. Evolution, 62, 2868–2883. https://doi.org/10.1111/j.1558-5646.2008.00482.x 18752605 10.1111/j.1558-5646.2008.00482.x

[ece34024-bib-0065] Weir, B. , & Cockerham, C. (1984). Estimating F‐statistics for the analysis of population structure. Evolution, 38, 1358–1370. 28563791 10.1111/j.1558-5646.1984.tb05657.x

[ece34024-bib-0066] Wellenreuther, M. , Sánchez‐Guillén, R. A. , Cordero‐Rivera, A. , & Hansson, B. (2010). Development of 12 polymorphic microsatellite loci in *Ischnura elegans* (Odonata: Coenagrionidae). Molecular Ecology Resources, 10, 576–579.21565062

[ece34024-bib-0067] Wellenreuther, M. , Sánchez‐Guillén, R. A. , Cordero‐Rivera, A. , Svensson, E. I. , & Hansson, B. (2011). Environmental and climatic determinants of molecular diversity and genetic population structure in a coenagrionid damselfly. PLoS ONE, 6, e20440 https://doi.org/10.1371/journal.pone.0020440 21655216 10.1371/journal.pone.0020440 PMC3105071

[ece34024-bib-0068] Wellenreuther, M. , Tynkkynen, K. , & Svensson, E. I. (2010). Simulating range expansion: Male species recognition and loss of premating isolation in damselflies. Evolution, 64, 242–252. https://doi.org/10.1111/j.1558-5646.2009.00815.x 19674095 10.1111/j.1558-5646.2009.00815.x

[ece34024-bib-0069] Whitney, K. D. , Randell, R. A. , & Rieseberg, L. H. (2006). Adaptive introgression of herbivore resistance traits in the weedy sunflower *Helianthus annuus* . The American Naturalist, 167, 794–807. https://doi.org/10.1086/504606 10.1086/504606 16649157

[ece34024-bib-0070] Zemanova, M. A. , Knop, E. , & Heckel, G. (2017). Introgressive replacement of natives by invading Arion pest slugs. Scientific Reports, 7, 14908 https://doi.org/10.1038/s41598-017-14619-y 29097725 10.1038/s41598-017-14619-y PMC5668256

