# Supplementary material for: Molecular and ecological signatures of an expanding hybrid zone
Source: Ecol Evol. 2018 Apr 16;8(10):4793–806. doi: 10.1002/ece3.4024 (PMC5980427; doi:10.1002/ece3.4024)
Supplement: Supplementary file 1 [file ECE3-8-4793-s001.docx]

# Supplementary Tables and Figures

## Supplementary Table 1. Results from genotypic linkage disequilibrium analyses of pairs of loci in each of three regions: allopatric *I. elegans* populations, sympatric populations, and allopatric *I. graellsii* populations.

| Region | Locus#A | Locus#B | P-value | S.E. | Analysed | p < 0.05 |
| --- | --- | --- | --- | --- | --- | --- |
| Allopatric *I. elegans* | Locus-1 | Locus-2 | 0.1973 | 0.0292 | 1 | 0 |
| Allopatric *I. elegans* | Locus-1 | Locus-3 | 0.9091 | 0.0270 | 1 | 0 |
| Allopatric *I. elegans* | Locus-2 | Locus-3 | 0.6444 | 0.0408 | 1 | 0 |
| Allopatric *I. elegans* | Locus-1 | Locus-4 | 0.9848 | 0.0094 | 1 | 0 |
| Allopatric *I. elegans* | Locus-2 | Locus-4 | 0.7387 | 0.0344 | 1 | 0 |
| Allopatric *I. elegans* | Locus-3 | Locus-4 | 0.8590 | 0.0313 | 1 | 0 |
| Allopatric *I. elegans* | Locus-1 | Locus-5 | 1.0000 | 0.0000 | 1 | 0 |
| Allopatric *I. elegans* | Locus-2 | Locus-5 | 1.0000 | 0.0000 | 1 | 0 |
| Allopatric *I. elegans* | Locus-3 | Locus-5 | 0.8028 | 0.0372 | 1 | 0 |
| Allopatric *I. elegans* | Locus-4 | Locus-5 | 0.2158 | 0.0394 | 1 | 0 |
| Allopatric *I. elegans* | Locus-1 | Locus-6 | NA |  | 0 | 0 |
| Allopatric *I. elegans* | Locus-2 | Locus-6 | NA |  | 0 | 0 |
| Allopatric *I. elegans* | Locus-3 | Locus-6 | NA |  | 0 | 0 |
| Allopatric *I. elegans* | Locus-4 | Locus-6 | NA |  | 0 | 0 |
| Allopatric *I. elegans* | Locus-5 | Locus-6 | NA |  | 0 | 0 |
| Allopatric *I. elegans* | Locus-1 | Locus-7 | 0.7042 | 0.0316 | 1 | 0 |
| Allopatric *I. elegans* | Locus-2 | Locus-7 | 0.3079 | 0.0312 | 1 | 0 |
| Allopatric *I. elegans* | Locus-3 | Locus-7 | 0.0325 | 0.0146 | 1 | 1 |
| Allopatric *I. elegans* | Locus-4 | Locus-7 | 0.9727 | 0.0085 | 1 | 0 |
| Allopatric *I. elegans* | Locus-5 | Locus-7 | 0.9986 | 0.0013 | 1 | 0 |
| Allopatric *I. elegans* | Locus-6 | Locus-7 | NA |  | 0 | 0 |
| Allopatric *I. elegans* | Locus-1 | Locus-8 | 0.4069 | 0.0418 | 1 | 0 |
| Allopatric *I. elegans* | Locus-2 | Locus-8 | 0.8214 | 0.0340 | 1 | 0 |
| Allopatric *I. elegans* | Locus-3 | Locus-8 | 0.8954 | 0.0296 | 1 | 0 |
| Allopatric *I. elegans* | Locus-4 | Locus-8 | 0.8602 | 0.0335 | 1 | 0 |
| Allopatric *I. elegans* | Locus-5 | Locus-8 | 0.1606 | 0.0335 | 1 | 0 |
| Allopatric *I. elegans* | Locus-6 | Locus-8 | NA |  | 0 | 0 |
| Allopatric *I. elegans* | Locus-7 | Locus-8 | 0.0285 | 0.0154 | 1 | 1 |
| Allopatric *I. elegans* | Locus-1 | Locus-9 | 0.1242 | 0.0224 | 1 | 0 |
| Allopatric *I. elegans* | Locus-2 | Locus-9 | 0.8850 | 0.0249 | 1 | 0 |
| Allopatric *I. elegans* | Locus-3 | Locus-9 | 0.7157 | 0.0413 | 1 | 0 |
| Allopatric *I. elegans* | Locus-4 | Locus-9 | 0.3018 | 0.0412 | 1 | 0 |
| Allopatric *I. elegans* | Locus-5 | Locus-9 | 0.5312 | 0.0466 | 1 | 0 |
| Allopatric *I. elegans* | Locus-6 | Locus-9 | NA |  | 0 | 0 |
| Allopatric *I. elegans* | Locus-7 | Locus-9 | 0.7072 | 0.0333 | 1 | 0 |
| Allopatric *I. elegans* | Locus-8 | Locus-9 | 0.1841 | 0.0352 | 1 | 0 |
| Allopatric *I. elegans* | Locus-1 | Locus-10 | 1.0000 | 0.0000 | 1 | 0 |
| Allopatric *I. elegans* | Locus-2 | Locus-10 | 1.0000 | 0.0000 | 1 | 0 |
| Allopatric *I. elegans* | Locus-3 | Locus-10 | 1.0000 | 0.0000 | 1 | 0 |
| Allopatric *I. elegans* | Locus-4 | Locus-10 | 0.0049 | 0.0049 | 1 | 1 |
| Allopatric *I. elegans* | Locus-5 | Locus-10 | 1.0000 | 0.0000 | 1 | 0 |
| Allopatric *I. elegans* | Locus-6 | Locus-10 | NA |  | 0 | 0 |
| Allopatric *I. elegans* | Locus-7 | Locus-10 | 0.7167 | 0.0354 | 1 | 0 |
| Allopatric *I. elegans* | Locus-8 | Locus-10 | 1.0000 | 0.0000 | 1 | 1 |
| Allopatric *I. elegans* | Locus-9 | Locus-10 | 0.6541 | 0.0447 | 1 | 0 |
| Allopatric *I. elegans* | Locus-1 | Locus-11 | 0.0762 | 0.0208 | 1 | 0 |
| Allopatric *I. elegans* | Locus-2 | Locus-11 | 0.1868 | 0.0318 | 1 | 0 |
| Allopatric *I. elegans* | Locus-3 | Locus-11 | 0.5450 | 0.0466 | 1 | 0 |
| Allopatric *I. elegans* | Locus-4 | Locus-11 | 0.9775 | 0.0132 | 1 | 0 |
| Allopatric *I. elegans* | Locus-5 | Locus-11 | 0.7265 | 0.0414 | 1 | 0 |
| Allopatric *I. elegans* | Locus-6 | Locus-11 | NA |  | 0 | 0 |
| Allopatric *I. elegans* | Locus-7 | Locus-11 | 0.7443 | 0.0328 | 1 | 0 |
| Allopatric *I. elegans* | Locus-8 | Locus-11 | 0.2736 | 0.0431 | 1 | 0 |
| Allopatric *I. elegans* | Locus-9 | Locus-11 | 0.9802 | 0.0124 | 1 | 0 |
| Allopatric *I. elegans* | Locus-10 | Locus-11 | 1.0000 | 0.0000 | 1 | 0 |
| Allopatric *I. elegans* | Locus-1 | Locus-12 | 0.3510 | 0.0252 | 1 | 0 |
| Allopatric *I. elegans* | Locus-2 | Locus-12 | 0.9771 | 0.0070 | 1 | 0 |
| Allopatric *I. elegans* | Locus-3 | Locus-12 | 0.9570 | 0.0151 | 1 | 0 |
| Allopatric *I. elegans* | Locus-4 | Locus-12 | 0.8712 | 0.0240 | 1 | 0 |
| Allopatric *I. elegans* | Locus-5 | Locus-12 | 0.9882 | 0.0067 | 1 | 0 |
| Allopatric *I. elegans* | Locus-6 | Locus-12 | NA |  | 0 | 0 |
| Allopatric *I. elegans* | Locus-7 | Locus-12 | 0.7547 | 0.0209 | 1 | 0 |
| Allopatric *I. elegans* | Locus-8 | Locus-12 | 0.1620 | 0.0283 | 1 | 0 |
| Allopatric *I. elegans* | Locus-9 | Locus-12 | 0.8751 | 0.0216 | 1 | 0 |
| Allopatric *I. elegans* | Locus-10 | Locus-12 | 0.7498 | 0.0336 | 1 | 0 |
| Allopatric *I. elegans* | Locus-11 | Locus-12 | 0.4609 | 0.0383 | 1 | 0 |
|  |  |  |  |  | *55* | *4* |
| Sympatric | Locus-1 | Locus-2 | 1.0000 | 0.0000 | 1 | 1 |
| Sympatric | Locus-1 | Locus-3 | 0.0289 | 0.0165 | 1 | 1 |
| Sympatric | Locus-2 | Locus-3 | 1.0000 | 0.0000 | 1 | 1 |
| Sympatric | Locus-1 | Locus-4 | 0.4380 | 0.0459 | 1 | 0 |
| Sympatric | Locus-2 | Locus-4 | 0.0390 | 0.0166 | 1 | 1 |
| Sympatric | Locus-3 | Locus-4 | 0.0169 | 0.0121 | 1 | 1 |
| Sympatric | Locus-1 | Locus-5 | 0.1426 | 0.0315 | 1 | 0 |
| Sympatric | Locus-2 | Locus-5 | 0.5517 | 0.0482 | 1 | 0 |
| Sympatric | Locus-3 | Locus-5 | 0.0004 | 0.0004 | 1 | 1 |
| Sympatric | Locus-4 | Locus-5 | 0.0585 | 0.0209 | 1 | 0 |
| Sympatric | Locus-1 | Locus-6 | 0.0007 | 0.0007 | 1 | 1 |
| Sympatric | Locus-2 | Locus-6 | 0.4875 | 0.0480 | 1 | 0 |
| Sympatric | Locus-3 | Locus-6 | 0.1005 | 0.0293 | 1 | 0 |
| Sympatric | Locus-4 | Locus-6 | 0.0035 | 0.0035 | 1 | 1 |
| Sympatric | Locus-5 | Locus-6 | 0.5164 | 0.0496 | 1 | 0 |
| Sympatric | Locus-1 | Locus-7 | 0.9517 | 0.0142 | 1 | 0 |
| Sympatric | Locus-2 | Locus-7 | 0.9240 | 0.0225 | 1 | 0 |
| Sympatric | Locus-3 | Locus-7 | 0.9657 | 0.0143 | 1 | 0 |
| Sympatric | Locus-4 | Locus-7 | 0.6441 | 0.0375 | 1 | 0 |
| Sympatric | Locus-5 | Locus-7 | 0.5303 | 0.0443 | 1 | 0 |
| Sympatric | Locus-6 | Locus-7 | 0.3677 | 0.0459 | 1 | 0 |
| Sympatric | Locus-1 | Locus-8 | 0.1358 | 0.0326 | 1 | 0 |
| Sympatric | Locus-2 | Locus-8 | 0.0390 | 0.0169 | 1 | 1 |
| Sympatric | Locus-3 | Locus-8 | 0.0938 | 0.0288 | 1 | 0 |
| Sympatric | Locus-4 | Locus-8 | 0.2228 | 0.0398 | 1 | 0 |
| Sympatric | Locus-5 | Locus-8 | 1.0000 | 0.0000 | 1 | 0 |
| Sympatric | Locus-6 | Locus-8 | 0.0847 | 0.0275 | 1 | 0 |
| Sympatric | Locus-7 | Locus-8 | 0.3396 | 0.0430 | 1 | 0 |
| Sympatric | Locus-1 | Locus-9 | 0.0703 | 0.0201 | 1 | 0 |
| Sympatric | Locus-2 | Locus-9 | 0.2018 | 0.0363 | 1 | 0 |
| Sympatric | Locus-3 | Locus-9 | 0.7284 | 0.0413 | 1 | 0 |
| Sympatric | Locus-4 | Locus-9 | 0.2001 | 0.0357 | 1 | 0 |
| Sympatric | Locus-5 | Locus-9 | 0.1591 | 0.0344 | 1 | 0 |
| Sympatric | Locus-6 | Locus-9 | 0.4924 | 0.0488 | 1 | 0 |
| Sympatric | Locus-7 | Locus-9 | 0.3966 | 0.0414 | 1 | 0 |
| Sympatric | Locus-8 | Locus-9 | 0.3105 | 0.0452 | 1 | 0 |
| Sympatric | Locus-1 | Locus-10 | 0.1252 | 0.0328 | 1 | 0 |
| Sympatric | Locus-2 | Locus-10 | 0.3964 | 0.0476 | 1 | 0 |
| Sympatric | Locus-3 | Locus-10 | 0.9972 | 0.0027 | 1 | 0 |
| Sympatric | Locus-4 | Locus-10 | 1.0000 | 0.0000 | 1 | 0 |
| Sympatric | Locus-5 | Locus-10 | 1.0000 | 0.0000 | 1 | 0 |
| Sympatric | Locus-6 | Locus-10 | 1.0000 | 0.0000 | 1 | 0 |
| Sympatric | Locus-7 | Locus-10 | 0.0331 | 0.0125 | 1 | 1 |
| Sympatric | Locus-8 | Locus-10 | 0.8063 | 0.0394 | 1 | 0 |
| Sympatric | Locus-9 | Locus-10 | 0.1891 | 0.0381 | 1 | 0 |
| Sympatric | Locus-1 | Locus-11 | 0.9191 | 0.0249 | 1 | 0 |
| Sympatric | Locus-2 | Locus-11 | 0.0840 | 0.0243 | 1 | 0 |
| Sympatric | Locus-3 | Locus-11 | 0.4151 | 0.0447 | 1 | 0 |
| Sympatric | Locus-4 | Locus-11 | 0.7195 | 0.0415 | 1 | 0 |
| Sympatric | Locus-5 | Locus-11 | 0.1486 | 0.0342 | 1 | 0 |
| Sympatric | Locus-6 | Locus-11 | 0.4912 | 0.0480 | 1 | 0 |
| Sympatric | Locus-7 | Locus-11 | 0.6397 | 0.0380 | 1 | 0 |
| Sympatric | Locus-8 | Locus-11 | 0.5085 | 0.0475 | 1 | 0 |
| Sympatric | Locus-9 | Locus-11 | 0.1698 | 0.0337 | 1 | 0 |
| Sympatric | Locus-10 | Locus-11 | 0.6908 | 0.0455 | 1 | 0 |
| Sympatric | Locus-1 | Locus-12 | 0.2882 | 0.0357 | 1 | 0 |
| Sympatric | Locus-2 | Locus-12 | 0.6464 | 0.0385 | 1 | 0 |
| Sympatric | Locus-3 | Locus-12 | 0.7778 | 0.0327 | 1 | 0 |
| Sympatric | Locus-4 | Locus-12 | 0.9674 | 0.0115 | 1 | 0 |
| Sympatric | Locus-5 | Locus-12 | 0.9357 | 0.0171 | 1 | 0 |
| Sympatric | Locus-6 | Locus-12 | 0.2888 | 0.0419 | 1 | 0 |
| Sympatric | Locus-7 | Locus-12 | 0.7359 | 0.0343 | 1 | 0 |
| Sympatric | Locus-8 | Locus-12 | 0.7355 | 0.0370 | 1 | 0 |
| Sympatric | Locus-9 | Locus-12 | 0.9893 | 0.0039 | 1 | 0 |
| Sympatric | Locus-10 | Locus-12 | 0.9019 | 0.0244 | 1 | 0 |
| Sympatric | Locus-11 | Locus-12 | 0.2682 | 0.0318 | 1 | 0 |
|  |  |  |  |  | *66* | *10* |
| Allopatric *I. graellsii* | Locus-1 | Locus-2 | NA |  | 0 | 0 |
| Allopatric *I. graellsii* | Locus-1 | Locus-3 | NA |  | 0 | 0 |
| Allopatric *I. graellsii* | Locus-2 | Locus-3 | 1.0000 | 0.0000 | 1 | 0 |
| Allopatric *I. graellsii* | Locus-1 | Locus-4 | NA |  | 0 | 0 |
| Allopatric *I. graellsii* | Locus-2 | Locus-4 | NA |  | 0 | 0 |
| Allopatric *I. graellsii* | Locus-3 | Locus-4 | NA |  | 0 | 0 |
| Allopatric *I. graellsii* | Locus-1 | Locus-5 | NA |  | 0 | 0 |
| Allopatric *I. graellsii* | Locus-2 | Locus-5 | NA |  | 0 | 0 |
| Allopatric *I. graellsii* | Locus-3 | Locus-5 | NA |  | 0 | 0 |
| Allopatric *I. graellsii* | Locus-4 | Locus-5 | NA |  | 1 | 0 |
| Allopatric *I. graellsii* | Locus-1 | Locus-6 | NA |  | 0 | 0 |
| Allopatric *I. graellsii* | Locus-2 | Locus-6 | NA |  | 0 | 0 |
| Allopatric *I. graellsii* | Locus-3 | Locus-6 | 0.4951 | 0.0036 | 1 | 0 |
| Allopatric *I. graellsii* | Locus-4 | Locus-6 | NA |  | 0 | 0 |
| Allopatric *I. graellsii* | Locus-5 | Locus-6 | NA |  | 0 | 0 |
| Allopatric *I. graellsii* | Locus-1 | Locus-7 | NA |  | 0 | 0 |
| Allopatric *I. graellsii* | Locus-2 | Locus-7 | 1.0000 | 0.0000 | 1 | 0 |
| Allopatric *I. graellsii* | Locus-3 | Locus-7 | 1.0000 | 0.0000 | 1 | 0 |
| Allopatric *I. graellsii* | Locus-4 | Locus-7 | NA |  | 0 | 0 |
| Allopatric *I. graellsii* | Locus-5 | Locus-7 | NA |  | 0 | 0 |
| Allopatric *I. graellsii* | Locus-6 | Locus-7 | 1.0000 | 0.0000 | 1 | 0 |
| Allopatric *I. graellsii* | Locus-1 | Locus-8 | NA |  | 0 | 0 |
| Allopatric *I. graellsii* | Locus-2 | Locus-8 | 1.0000 | 0.0000 | 1 | 0 |
| Allopatric *I. graellsii* | Locus-3 | Locus-8 | 0.2311 | 0.0173 | 1 | 0 |
| Allopatric *I. graellsii* | Locus-4 | Locus-8 | NA |  | 0 | 0 |
| Allopatric *I. graellsii* | Locus-5 | Locus-8 | NA |  | 0 | 0 |
| Allopatric *I. graellsii* | Locus-6 | Locus-8 | 1.0000 | 0.0000 | 1 | 0 |
| Allopatric *I. graellsii* | Locus-7 | Locus-8 | 0.1429 | 0.0182 | 1 | 0 |
| Allopatric *I. graellsii* | Locus-1 | Locus-9 | NA |  | 0 | 0 |
| Allopatric *I. graellsii* | Locus-2 | Locus-9 | NA |  | 0 | 0 |
| Allopatric *I. graellsii* | Locus-3 | Locus-9 | 1.0000 | 0.0000 | 1 | 0 |
| Allopatric *I. graellsii* | Locus-4 | Locus-9 | NA |  | 0 | 0 |
| Allopatric *I. graellsii* | Locus-5 | Locus-9 | NA |  | 0 | 0 |
| Allopatric *I. graellsii* | Locus-6 | Locus-9 | NA |  | 0 | 0 |
| Allopatric *I. graellsii* | Locus-7 | Locus-9 | 0.0632 | 0.0044 | 1 | 0 |
| Allopatric *I. graellsii* | Locus-8 | Locus-9 | 0.5342 | 0.0251 | 1 | 0 |
| Allopatric *I. graellsii* | Locus-1 | Locus-10 | NA |  | 0 | 0 |
| Allopatric *I. graellsii* | Locus-2 | Locus-10 | NA |  | 0 | 0 |
| Allopatric *I. graellsii* | Locus-3 | Locus-10 | 0.6692 | 0.0087 | 1 | 0 |
| Allopatric *I. graellsii* | Locus-4 | Locus-10 | NA |  | 0 | 0 |
| Allopatric *I. graellsii* | Locus-5 | Locus-10 | NA |  | 0 | 0 |
| Allopatric *I. graellsii* | Locus-6 | Locus-10 | NA |  | 0 | 0 |
| Allopatric *I. graellsii* | Locus-7 | Locus-10 | 1.0000 | 0.0000 | 1 | 0 |
| Allopatric *I. graellsii* | Locus-8 | Locus-10 | 0.6744 | 0.0261 | 1 | 0 |
| Allopatric *I. graellsii* | Locus-9 | Locus-10 | 1.0000 | 0.0000 | 1 | 0 |
| Allopatric *I. graellsii* | Locus-1 | Locus-11 | NA |  | 1 | 0 |
| Allopatric *I. graellsii* | Locus-2 | Locus-11 | NA |  | 0 | 0 |
| Allopatric *I. graellsii* | Locus-3 | Locus-11 | NA |  | 0 | 0 |
| Allopatric *I. graellsii* | Locus-4 | Locus-11 | NA |  | 0 | 0 |
| Allopatric *I. graellsii* | Locus-5 | Locus-11 | No dat |  | 1 | 0 |
| Allopatric *I. graellsii* | Locus-6 | Locus-11 | No dat |  | 1 | 0 |
| Allopatric *I. graellsii* | Locus-7 | Locus-11 | NA |  | 0 | 0 |
| Allopatric *I. graellsii* | Locus-8 | Locus-11 | 1.0000 | 0.0000 | 1 | 0 |
| Allopatric *I. graellsii* | Locus-9 | Locus-11 | 1.0000 | 0.0000 | 1 | 0 |
| Allopatric *I. graellsii* | Locus-10 | Locus-11 | NA |  | 0 | 0 |
| Allopatric *I. graellsii* | Locus-1 | Locus-12 | NA |  | 0 | 0 |
| Allopatric *I. graellsii* | Locus-2 | Locus-12 | NA |  | 0 | 0 |
| Allopatric *I. graellsii* | Locus-3 | Locus-12 | 1.0000 | 0.0000 | 1 | 0 |
| Allopatric *I. graellsii* | Locus-4 | Locus-12 | NA |  | 0 | 0 |
| Allopatric *I. graellsii* | Locus-5 | Locus-12 | NA |  | 0 | 0 |
| Allopatric *I. graellsii* | Locus-6 | Locus-12 | NA |  | 0 | 0 |
| Allopatric *I. graellsii* | Locus-7 | Locus-12 | 1.0000 | 0.0000 | 1 | 0 |
| Allopatric *I. graellsii* | Locus-8 | Locus-12 | 1.0000 | 0.0000 | 1 | 0 |
| Allopatric *I. graellsii* | Locus-9 | Locus-12 | NA |  | 0 | 0 |
| Allopatric *I. graellsii* | Locus-10 | Locus-12 | NA |  | 0 | 0 |
| Allopatric *I. graellsii* | Locus-11 | Locus-12 | NA |  | 0 | 0 |
|  |  |  |  |  | *25* | *0* |
